# Kinetic Analysis of Growth Activity in Enlarging Papillary Thyroid Microcarcinomas

**DOI:** 10.1089/thy.2019.0396

**Published:** 2019-12-16

**Authors:** Yasuhiro Ito, Akira Miyauchi, Takumi Kudo, Takuya Higashiyama, Hiroo Masuoka, Minoru Kihara, Akihiro Miya

**Affiliations:** ^1^ Department of Surgery, Kuma Hospital, Kobe, Japan.; ^2^ Department of Internal Medicine, Kuma Hospital, Kobe, Japan.

**Keywords:** papillary microcarcinoma, thyroid, growth activity, kinetic analysis, thyroid cancer

## Abstract

***Background:*** Although papillary thyroid microcarcinoma (PMC) is generally stable on active surveillance, conversion surgery is recommended for enlarging tumors. However, it remains unclear which enlargement threshold should be considered sufficient to trigger surgery. This study analyzed changes in the growth activity of PMC, before and after enlargement.

***Methods:*** We enrolled 824 patients with PMC, in whom active surveillance was initiated between 2005 and 2011 (median duration of follow-up: 6.04 years). Changes in the maximal tumor size and tumor volume were evaluated. Point of enlargement (PE) was defined as the time at which maximal tumor size or tumor volume had increased by ≥3 mm (PE-M) or by ≥50% (PE-V), respectively. In patients with PMC who underwent at least three ultrasound examinations during the study period, we compared the tumor doubling rates (TDRs, designated as the inverse of doubling time) between pre- and post-PEs.

***Results:*** Ten-year enlargement-free survival rates based on maximal tumor size and tumor volume were 86.9% and 54.9%, respectively. The median post-PE TDRs was significantly lower than that of pre-PEs (−0.091/year vs. 0.509/year [*p* < 0.001] for PE-M, and −0.058/year vs. 0.370/year [*p* < 0.001] for PE-V), indicating decreased tumor growth after PEs. After PE-M and PE-V, the PMCs continued to rapidly enlarge (TDR >0.5/year) in only 6 (7.7%) and 11 (3.8%) patients and moderately enlarge (TDR 0.1–0.5/year) in 10 (12.8%) and 35 (12.1%) patients, respectively. Conversely, tumors shrank (TDR < −0.1/year) in 37 (47.4%) and 105 (36.1%) patients, respectively, and remained stable (TDR ranged between 0.1/year and −0.1/year) in 25 (32.1%) and 140 (48.1%) patients, respectively.

***Conclusion:*** Since most PMCs demonstrate a significant decrease in growth activity after enlargement, performing surgery immediately after the PE may be premature.

## Introduction

Papillary thyroid carcinomas measuring 10 mm or less are known as papillary thyroid microcarcinomas (PMCs). In recent years, guidelines published by the Japan Association of Endocrine Surgeons/Japanese Society of Thyroid Surgery (the present Japan Association of Endocrine Surgery) (1) and the American Thyroid Association (2) have recommended active surveillance for low-risk PMCs without high-risk features, such as clinical nodal metastases, distant metastases, or significant extrathyroidal extension to the adjacent organs. Favorable outcomes have been reported in patients with PMC who underwent active surveillance at Japanese institutions, such as at the Kuma Hospital and the Cancer Institute Hospital (Tokyo, Japan) (3–8). At present, results from a few prospective and retrospective studies are available from countries other than Japan, including the United States, South Korea, and Colombia (9–11).

Although most PMCs demonstrate no or very slow growth, some cases progress with an enlargement in tumor size, appearance of lymph node metastases, or both, and subsequently require conversion surgery. The evaluation of growth is a major issue for considering conversion surgery in patients undergoing active surveillance for PMC. We routinely measure two, or if possible, three dimensions of the PMC using ultrasound. However, we usually adopt only the greatest dimension (maximal tumor size) for evaluation. A growth of at least 3 mm in maximal tumor size is considered PMC enlargement at Kuma Hospital and at the Cancer Institute Hospital (3–8). In contrast, researchers in the United States and South Korea defined tumor progression (or growth) as an increase of at least 50% in tumor volume (9,10). Studies using this measure claim that evaluation based on tumor volume is able to identify progressing tumors earlier and more accurately.

Whether and how the growth activity of PMC changes after enlargement remains unclear. Immediate conversion surgery may be appropriate or mandatory in PMCs that continue to grow after enlargement. However, we have previously demonstrated that the rates of progression in PMCs decreased significantly with increasing age; we also found that 17% of PMCs shrank during active surveillance (3). Miyauchi *et al.* reported that the lifetime probability of disease progression in patients with PMC decreased significantly with increasing age at presentation (12). They also hypothesized that the progression of PMCs is much more rapid before presentation than after and that the deceleration of growth rate is a common phenomenon in the natural course of PMCs (13). These findings strongly suggest that despite enlargement, the growth activity of PMCs may decrease or even disappear thereafter; tumors have also been found to shrink in certain cases. If this assumption is correct, conversion surgery immediately after tumor enlargement would result in overtreatment for many patients. Therefore, in this study, we investigated the change in the growth activity of PMCs after they were deemed to have enlarged.

## Materials and Methods

This study enrolled 824 patients with PMC, comprising 97 males and 727 females, with ages ranging from 20 to 83 (median: 58) years, who were enrolled in active surveillance between 2005 and 2011 (median duration of follow-up: 6.04 years, range: 1.11–12.7 years). All patients were diagnosed with papillary thyroid carcinoma by cytology and active surveillance was performed using ultrasound at least once per year. For each examination, the maximum diameter (*D*1) and the diameter in the direction perpendicular to the maximum diameter (*D*2) were measured. Tumor depth (*D*3) was often not a reliable measure due to ultrasound shadowing. Tumor volume (*V*) was then calculated using the ellipsoid equation: *π*/6 × *D*1 × *D*2 × *D*2 or *D*3. Tumor size or tumor volume was evaluated via serial ultrasound examinations, using the maximal tumor size to evaluate changes in size; two- or three-dimensional tumor volume measurements were used to evaluate changes in volume. The point of enlargement (PE) was defined as time at which maximal tumor size or tumor volume increased by ≥3 mm (PE-M) or by ≥50% (PE-V), respectively. Patients with an increase in maximal tumor size of ≥3 mm were informed of tumor enlargement and surgery was recommended. However, in certain cases, active surveillance after enlargement was continued per the patients' request until the tumor reached 13 mm in size. Increases of ≥50% in the tumor volume appeared earlier than the threshold increases in tumor size. In our practice, a ≥50% increase in tumor volume was not regarded as the optimal time for conversion surgery. In patients who underwent at least three ultrasound evaluations, we calculated the tumor doubling time (TDT) based on a previously described formula (13). Time (*T*) was set as the time interval between presentation and measurement and was also calculated using the Doubling Time & Progression Calculator.

$$\begin{array}{c} a=\left(n\sum_{k=1}^{n}T_{k}\times \log\left(V_{k}\right)-\sum_{k=1}^{n}T_{k}\times \sum_{k=1}^{n}\log V_{k}\right)\\ /\left(\sum_{k=1}^{n}{T^{2}}_{k}-{\left(\sum_{k=1}^{n}T_{k}\right)}^{2}\right). \end{array}$$

However, there were cases of PMCs that did not grow or even shrank, causing discontinuity among the positive and negative TDT values. To address this, we used the inverse of the TDT, which was designated as the tumor doubling rate (TDR) (13) since this value indicates the number of doublings per unit time.

Time-sequence-based data were analyzed using the Kaplan–Meier analysis and statistically compared using the log-rank test. Between groups, differences were analyzed using the Mann–Whitney *U* and Wilcoxon signed-rank tests for skewed and paired skewed variables, respectively. All statistical analyses were conducted using the StatFlex version 6.0 software package. A *p*-value of less than 0.05 was considered to be statistically significant.

## Results

### Tumor enlargement patient outcomes

**FIG. 1.**
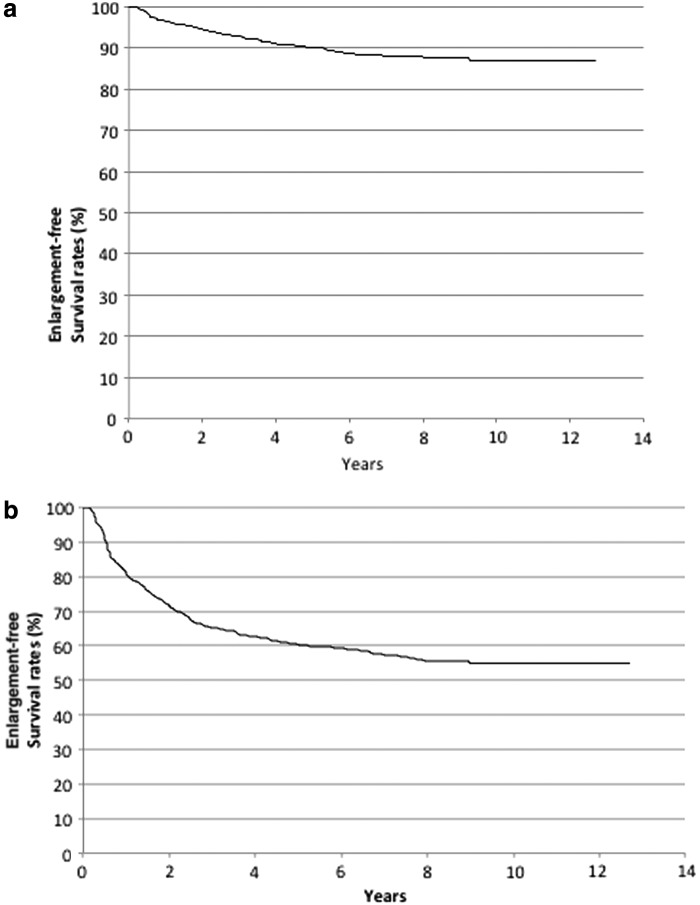
(**a**) Kaplan–Meier curves of tumor enlargement-free survival in PMC cases subjected to PE-M. (**b**) Kaplan–Meier curves of tumor enlargement-free survival in PMC cases subjected to PE-V. PMC, papillary thyroid microcarcinoma.

Tumor enlargement was defined in this study as an increase in the maximal tumor size or tumor volume of ≥3 mm (PE-M) or ≥50% (PE-V), respectively. Among the 824 patients enrolled in this study, 92 and 333 attained PE-M and PE-V status, respectively. Figure 1a and b demonstrate the Kaplan–Meier curves of tumor enlargement based on maximal tumor size and tumor volume in this cohort, which showed that the 10-year enlargement-free survival rates were 86.7% and 54.9%, respectively. Eighty-three patients underwent surgery, and among these patients, surgery was performed at the pre-PE-M phase in 60 cases, at the post-PE-M phase in 23 cases, at the pre-PE-V phase in 39 cases, and at the post-PE-V phase in 44 cases. During the time period of active surveillance following conversion surgery, distant metastases, recurrences, or deaths resulting from thyroid carcinoma were not observed in any of the cases.

### Comparison of TDRs between pre- and post-PEs

**FIG. 2.**
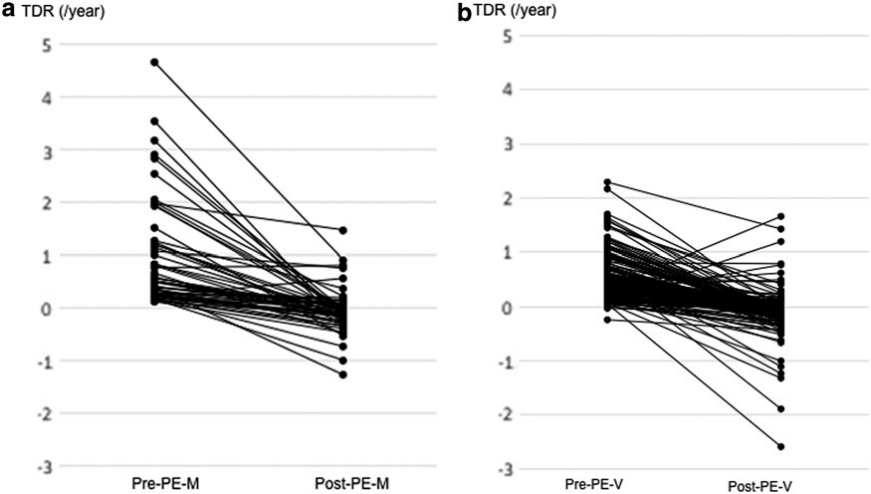
(**a**) Changes in PMC TDR in pre- and post-PE-M cases (*n* = 56). (**b**) Changes in PMC TDR in pre- and post-PE-V cases (*n* = 163). TDR, tumor doubling rate.

We subsequently compared the pre- and post-PE TDRs in patients who underwent at least three ultrasound evaluations during the study period. TDRs were separately evaluated in 69 and 78 patients in the pre- and post-PE-M phases, respectively. The median TDR in the post-PE-M cases was significantly lower (*p* < 0.001) than that of pre-PE-M cases (−0.0907/year vs. 0.5088/year), indicating a decrease in tumor growth rates of PMCs that grew by ≥3 mm. TDRs measured within individual cases across the pre- and post-PE-M phases were evaluated in 56 patients, and the changes in pre- and post-PE-M TDRs for these cases are shown in Figure 2a. Among the 56 cases analyzed, the post-PE-M TDRs were lower in 54 (96%) cases and higher in 2 (4%) cases when compared with the pre-PE-M phase.

The pre- and post-PE-V TDRs were separately evaluated in 197 and 291 patients, respectively. The median post-PE-V TDR was also significantly lower (*p* < 0.001) than that of the pre-PE-V phase (−0.058/year vs. 0.370/year), indicating a decrease in tumor growth rates after tumor volumes increased ≥50%. TDRs measured within individual cases across the pre- and post-PE-V phases were evaluated in 163 patients, and the changes between these phases are shown in Figure 2b. Among the 163 cases analyzed, the post-PE-V TDR were lower in 156 (96%) cases and higher in 7 (4%) cases when compared with the pre-PE-V phase.

### Patient age stratified subset analysis of TDR

**FIG. 3.**
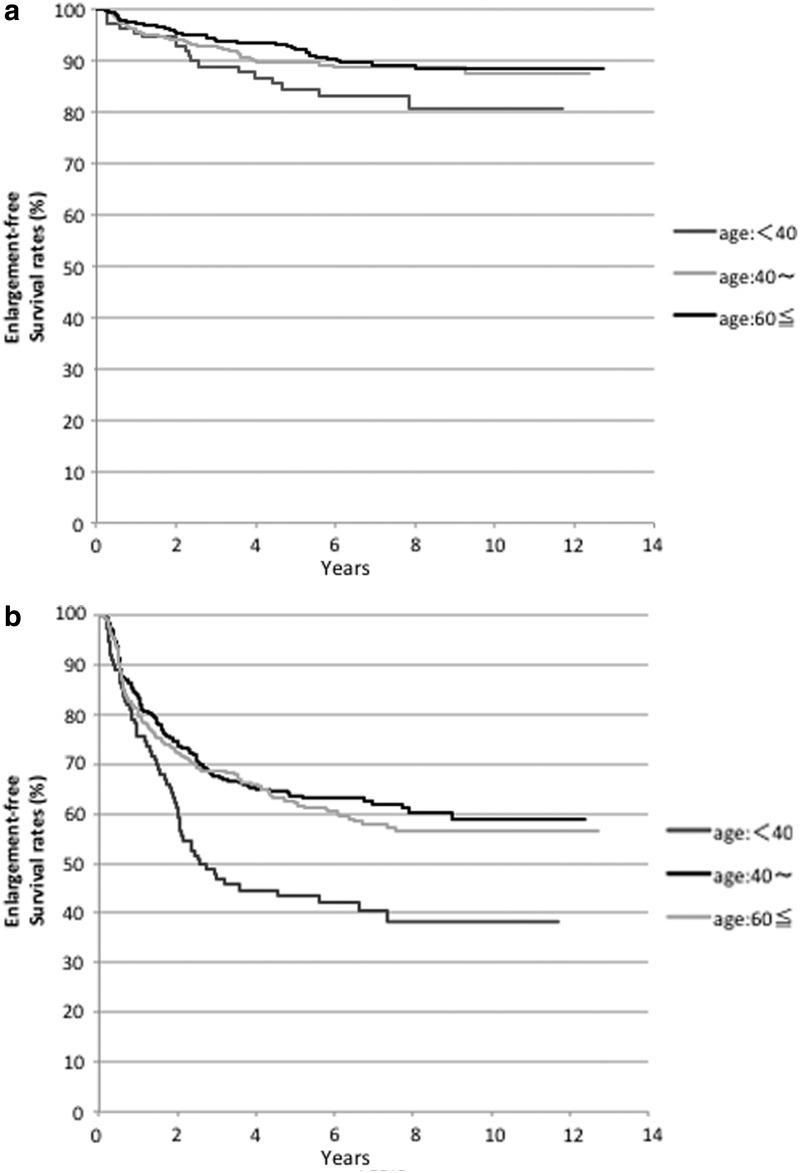
(**a**) Stratified Kaplan–Meier curves of tumor enlargement-free survival in PMC cases subjected to PE-M in patients aged <40 years, between 40 and 59 years, and ≥60 years. **(b)** Stratified Kaplan–Meier curves of tumor enlargement-free survival in PMC cases subjected to PE-V in patients aged <40 years, between 40 and 59 years, and ≥60 years.

**FIG. 4.**
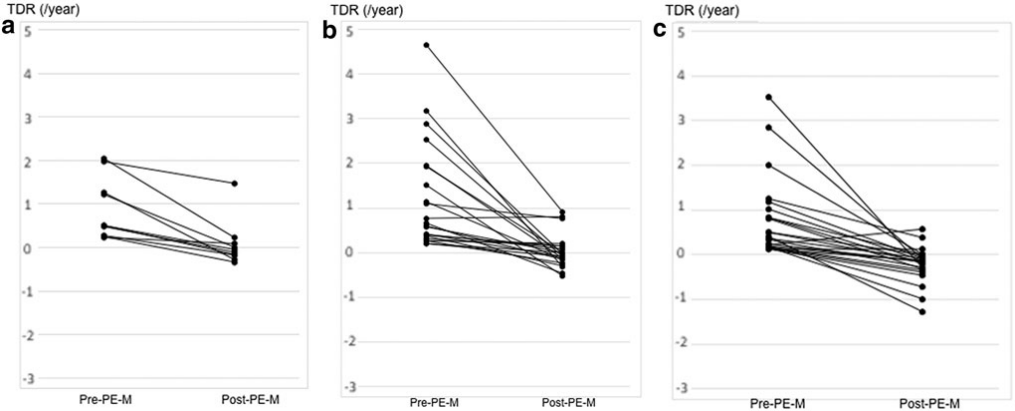
(**a**) Changes in PMC TDR of patients aged <40 years in pre- and post-PE-M cases (*n* = 10). (**b**) Changes in PMC TDR of patients aged between 40 and 59 years in pre- and post-PE-M cases (*n* = 22). (**c**) Changes in PMC TDR of patients aged ≥60 years in pre- and post-PE-M cases (*n* = 24).

**FIG. 5.**
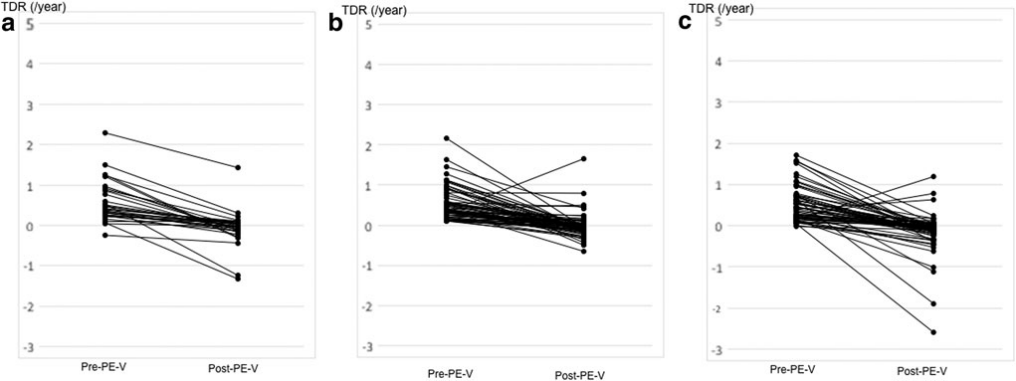
(**a**) Changes in PMC TDR of patients aged <40 years in pre- and post-PE-V cases (*n* = 32). (**b**) Changes in PMC TDR of patients aged between 40 and 59 years in pre- and post-PE-V cases (*n* = 67). (**c**) Changes in PMC TDR of patients aged ≥60 years in pre- and post-PE-V cases (*n* = 64).

We also performed subset analyses based on the age of patients at time of presentation. Patients were stratified into three subgroups, which included those aged <40 years (*n* = 110), aged between 40 and 59 years (*n* = 348), and aged ≥60 years (*n* = 366). Figure 3a shows the Kaplan–Meier curves for the three age subgroups using maximal tumor size to define enlargement. The 10-year maximal tumor size-based enlargement-free survival rates were 80.6%, 87.4%, and 88.3% in patients aged <40, 40–59, and ≥60 years, respectively. The PMCs in patients aged <40 years were more likely to increase in size compared with two subgroups, although the difference did not attain statistical significance (*p* = 0.0503). Figure 3b shows the Kaplan–Meier curves for the three age subgroups using tumor volume to define enlargement. The 10-year enlargement-free survival rates in patients aged <40, 40–59, and ≥60 years were 38.3%, 58.9%, and 56.5%, respectively. The enlargement-free survival based on tumor volume in patients aged <40 years was significantly (*p* < 0.001) lower than that of the other two age subgroups. The median post-PE-M TDR in each subgroup was significantly lower than that of the pre-PE-M phase (*p* = 0.002: −0.056/year vs. 0.512/year in patients aged <40 years, *p* < 0.001: −0.025/year vs. 0.549/year in those aged 40–59 years, and *p* < 0.001: −0.142/year vs. 0.381/year in those aged ≥60 years). The median post-PE-V TDR in each subgroup was also significantly lower than that of the pre-PE-V phase (*p* < 0.001: −0.024/year vs. 0.321/year in patients aged <40 years, *p* < 0.001: −0.061/year vs. 0.384/year in those aged 40–59 years, and *p* < 0.001: −0.070/year vs. 0.350/year in those aged ≥60 years). Figures 4 and 5 describe the changes in TDRs between the pre- and post-PE phases in cases that could be evaluated across both phases. The post-PE-M TDRs in two patients, aged 40–59 and ≥60 years, respectively, were higher than those of the pre-PE-M phase. However, in all other patients, the post-PE-M TDRs were lower than those of the pre-PE-M phase. Compared with the pre-PE-V phase, the post-PE-V TDRs increased in one, three, and three patients aged <40, 40–59, and ≥60 years, respectively. All other cases showed a decrease in post-PE-V TDR relative to pre-PE-M regardless of age stratification.

### Changes in growth activity based on pre- and post-PE TDRs

The growth activity based on pre- and post-PE TDRs in the present study is shown in Table 1. Only in 6 (7.7%) and 11 (3.8%) patients, PMCs continued to enlarge rapidly (TDR >0.5/year) after reaching PE-M and PE-V, respectively. Conversely, 37 (47.4%) and 105 (36.1%) PMCs decreased in size (TDR < −0.1/year), and 25 (32.1%) and 142 (48.1%) PMCs remained stable (TDR between 0.1/year and −0.1/year), after reaching PE-M and PE-V, respectively. The PMCs in 25 (32.1%) and 142 (48.1%) patients were stable with post-PE-M and post-PE-V TDRs ranging between 0.1/year and −0.1/year, respectively. The pre-PE-M and pre-PE-V TDRs were significantly higher than those of the post-PE-M and post-PE-V phases (*p* < 0.001 for both PE-M and PE-V). Table 2 demonstrates the pre- and post-PE-M TDRs in the three age subgroups. The pre-PE-V TDRs in each subset were significantly higher than those of the post-PE-M phase (*p* < 0.001 in all three age subgroups). However, the pre- and post-PE-V TDRs did not differ among the three age subgroups as shown in Table 3. In each subset, the pre-PE-V TDRs were significantly higher than those of the post-PE-V phase (*p* < 0.001 in all three age subgroups). However, the pre- and post-PE-V TDRs were similar among all the three age subgroups.

**Table 1.** Tumor Doubling Rates in Patients Who Underwent Pre- and Post-Point of Enlargement Active Surveillance

	No. of patients	Tumor doubling rate (per year)			
		>0.5,* n *(%)	0.1–0.5,* n *(%)	−0.1 to 0.1,* n *(%)	<−0.1,* n *(%)
Pre-PE-M	69	35 (50.8)	32 (46.4)	1 (1.4)	1 (1.4)
Post-PE-M	78	6 (7.7)	10 (12.8)	25 (32.1)	37 (47.4)
Pre-PE-V	197	77 (39.1)	99 (50.2)	20 (10.2)	1 (0.5)
Post-PE-V	291	11 (3.8)	35 (12.1)	140 (48.1)	105 (36.1)

PE, point of enlargement; PE-M, time at which maximal tumor size increased by ≥3 mm; PE-V, time at which tumor volume increased by ≥50%. Results of statistical analysis for pre-PE-M versus post-PE-M: *p* < 0.001, and pre-PE-V versus post PE-V: *p* < 0.001.

**Table 2.** Tumor Doubling Rates in Patients Who Underwent Active Surveillance Pre- and Post-PE-M in Stratified Based on Patient Age at Time of Presentation

		No. of patients	Tumor doubling rate (per year)			
			>0.5,* n *(%)	0.1–0.5,* n *(%)	−0.1 to 0.1,* n *(%)	<−0.1,* n *(%)
Pre-PE-M	<40 Years	12	7 (58.3)	5 (41.7)	0	0
Post-PE-M	<40 Years	15	1 (6.7)	2 (13.3)	5 (33.3)	7 (46.7)
Pre-PE-M	40–59 Years	29	15 (51.7)	13 (44.8)	0	1 (3.5)
Post-PE-M	40–59 Years	31	4 (12.9)	5 (16.1)	11 (35.5)	11 (35.5)
Pre-PE-M	≥60 Years	28	13 (46.5)	14 (50.0)	1 (0.5)	0
Post-PE-M	≥60 Years	32	1 (3.1)	3 (9.4)	9 (28.1)	19 (59.4)

Results of statistical analysis for pre-PE-M versus post-PE-M: *p* < 0.001 (≤40 years), *p* < 0.001 (41–59 years), *p* < 0.001 (≥60 years). Pre: *p* = 0.918 (≤40 years vs. 41–59 years), *p* = 0.539 (41–59 years vs. ≥60 years). Post: *p* = 0.861 (≤40 years vs. 41–59 years), *p* = 0.202 (41–59 years vs. ≥60 years).

**Table 3.** Tumor Doubling Rates in Patients Who Underwent Active Surveillance Pre- and Post-PE-V Stratified Based on Patient Age at Time of Presentation

			Tumor doubling rate (per year)			
		No. of patients	>0.5,* n *(%)	0.1–0.5,* n *(%)	−0.1 to 0.1,* n *(%)	<−0.1,* n *(%)
Pre-PE-V	<40 Years	39	13 (33.3)	21 (53.8)	4 (10.3)	1 (2.6)
Post-PE-V	<40 Years	54	2 (3.7)	10 (18.5)	27 (50.0)	15 (27.8)
Pre-PE-V	40–59 Years	83	34 (41.0)	43 (51.8)	6 (7.2)	0
Post-PE-V	40–59 Years	111	5 (4.6)	15 (13.5)	52 (46.8)	39 (35.1)
Pre-PE-V	≥60 Years	75	30 (40.0)	35 (46.7)	10 (13.3)	0
Post-PE-V	≥60 Years	126	4 (3.2)	10 (7.9)	61 (48.4)	51 (40.5)

Results of statistical analysis for pre-PE-V versus post-PE-V: *p* < 0.001 (≤40 years), *p* < 0.001 (41–59 years), *p* < 0.001 (≥60 years). Pre: *p* = 0.416 (≤40 years vs. 41–59 years), *p* = 0.644 (41–59 years vs. ≥60 years). Post: *p* = 0.722 (≤40 years vs. 41–59 years), *p* = 0.615 (41–59 years vs. ≥60 years).

## Discussion

The findings from this study demonstrate several interesting aspects of tumor growth in PMC. First, the 10-year enlargement-free survival rate based on the maximal tumor size was 86.7%, which is significantly higher than the rate calculated using tumor volume (54.9%). Second, irrespective of patient age, the pre-PE-M and pre-PE-V TDRs were significantly higher than those measured in the post-PE-M and post-PE-V phases. Third, following PE-M and PE-V, PMCs continued to rapidly grow (TDR >0.5/year) in only 6 (7.7%) and 11 (3.8%) patients and continued to moderately grow (TDR 0.1–0.5/year) in only 10 (12.8%) and 35 (12.1%) patients, respectively.

Compared with the 10-year PMC enlargement rate of 8% reported in our previous study, the rate in the present cohort was higher, at 13.3% (3). This may be attributed to the relatively smaller number of patients enrolled in this cohort (824 patients in the current study compared with 1235 patients in the previous study). However, the periods of active surveillance in both studies were similar.

Researchers from both the United States and South Korea claim that for PMCs tumor volume-based evaluation is superior since it is more sensitive to growth. Tuttle *et al.* reported that following 5 years of follow-up, the cumulative incidence of a greater than 50% increase in tumor volume was 24.8%; in contrast, only 12.1% experienced an increase in the maximal tumor size by ≥3 mm during the same period (9). In the present study, we also found that tumor volume-based 10-year enlargement-free survival rates were lower than those based on maximal tumor size. It may appear that evaluation on the basis of tumor volume is superior to evaluations based on maximal tumor size since PMCs with rapid growth activity were apparently detected earlier. However, there are certain pertinent issues in this evaluation. First, since two- or three-dimensional measurements are required, tumor volume may be subjected to considerably higher interobserver variation compared with maximal tumor diameter. Second, since a 50% increase in tumor volume defines PE, a PMC tumor measuring 6 mm × 6 mm × 6 mm increases in volume by 59% when it enlarges to 7 mm × 7 mm × 7 mm and is subsequently classified as an enlarged carcinoma. This judgment may be overly sensitive since we notably found that the growth activity of PMCs often decreased, or even regressed over time. Although PMCs grew during the initial stages of active surveillance, the incidence of a decrease in growth activity, or regression thereafter, was high. Ito *et al.* reported that PMCs are more likely to grow in patients aged ≤40 years. However, growth was reduced with advanced age (3). Miyauchi *et al.* estimated the lifetime probability of PMC progression using the age-decade-specific disease progression rate and found that 51% and 75% of patients in the third and fourth decades of life, respectively, do not require surgery in their lifetimes (12). The estimated probability of disease progression was reduced further with advancing age. Miyauchi *et al.* also reported that TDRs in patients with PMC aged ≤40 years were higher than that of older patients; in addition, in patients aged >60 years, the tumors were either stable or shrank more often than in younger patients (13). Miyauchi *et al.* also calculated the hypothetical TDR using tumor size and age at presentation, assuming that a single cancer cell (10 μm in diameter) was present at birth and grew at a constant rate until presentation. This value indicates the lowest growth rate for a single cancer cell to attain the size at presentation. They demonstrated that the calculated hypothetical TDRs before presentation (0.4–1.1/year, median: 0.5/year) were significantly higher than the actual TDRs observed during active surveillance (−12.8–1.32/year, median 0.0/year) (13). These data strongly suggest that PMCs grow rather rapidly in the initial stages of progression. However, in many cases, the growth decreases or even vanishes at certain time points. Additionally, we noted that the incidence of tumor shrinkage during active surveillance was high. Therefore, in patients undergoing active surveillance, a decision in favor of surgery in the initial phases of PMC growth is most likely to increase the likelihood of unnecessary surgery.

In this study, we investigated whether and how tumor growth activity changed after establishing tumor enlargement, based on either maximal tumor diameter ≥3 mm (PE-M) or tumor volume >50% (PE-V). The pre-PE-M and pre-PE-V TDRs were significantly higher than those of the post-PE-M and post-PE-V phases. After PE-M, 7.7% of PMCs showed rather rapid and continuous growth (TDR >0.5/year). However, 32.1% of tumors stabilized and 47.4% shrank. In addition, after PE-V, 3.7% tumors grew rather rapidly, but 48.1% stabilized and 36.1% shrank. These findings suggest that although a proportion of PMCs grow continuously, the growth activity of most PMCs stabilize or even decrease after enlargement. In all three age subgroups, the post-PE-M and post-PE-V TDRs were significantly lower than those of the pre-PE-M and pre-PE-V phases. These findings suggest that regardless of patient age, most PMCs decrease or stabilize in size and/or volume after reaching PE.

This phenomenon is well known in population dynamics where it is called carrying capacity, and it supports the evolutionary perspective to cancer initiation, promotion, and progression (14). Furthermore, it has also been observed in studies using preclinical models. Juan *et al.* showed that BRAF^V600E^-induced lung tumors in mice undergo senescence-like proliferative arrest when encountering an insufficiency of WNT/beta-catenin/c-MYC signaling (15). These findings suggest that additional escape mechanisms are required to overcome carcinoma growth constraints. Indeed, Choi *et al.* demonstrated that FoxM1, a component downstream of the AKT signaling pathway, overcomes oncogene-induced senescence in cells activated by BRAF^V600E^ and AKT signaling (16).

The recommendation and timing of conversion surgery is an important issue. As discussed previously, recommending surgery when tumor volume increases by >50% may be inappropriately early. This issue may be addressed by increasing the cutoff values for the increase in tumor volume. However, it is difficult to calculate tumor volumes and TDRs in the clinic on a real-time basis. Investigators at Kuma Hospital and the Cancer Institute Hospital have evaluated changes in the maximal tumor size during active surveillance of PMC; an enlargement in size of ≥3 mm was set as the criterion for PMC progression (3–8). When patients present to our hospital, they are informed about changes in tumor size or enlargement and surgical treatments are performed at the request of the patient. However, in cases that did not undergo initial surgery, we continued active surveillance until the tumor size exceeded 13 mm. Although this strategy may appear similar to the evaluation based on tumor volume, it is much simpler and is less likely to be affected by interobserver variations. Moreover, none of the patients in these two high-volume institutions who underwent conversion surgery based on these criteria experienced life-threatening recurrences or death from thyroid carcinoma. The findings of the study presented here should provoke further investigations into whether immediate conversion surgery when tumor size enlargement is ≥3 mm is truly an appropriate strategy for the management of PMC. In our cohort, even after an enlargement by ≥3 mm, some PMCs stabilized (32.1%) and some shrank (47.4%) (Table 1). It is also known that tumor enlargement is less likely to occur in older patients. In view of these findings, it may be appropriate to decide on the timing of conversion surgery in PMCs based on the change in size after enlargement. If tumor growth continues, conversion surgery should be considered. In other cases, continuous active surveillance is acceptable in anticipation of a possible cessation of tumor growth and/or tumor shrinkage. It may appear that this strategy simply postpones surgery that will be necessary at some point later in the patient's lifetime. However, considering the decrease in growth activity of PMCs with age, and after the PE (3,10,11), these patients may not require surgery altogether. In recent years, we continued active surveillance in enlarged PMCs until the tumor size reached ∼13 mm in patients who agreed to do so. It remains unclear whether this strategy for active surveillance is optimal and future studies are required to explicitly test this.

PMC progression includes not only enlargement of primary lesions but also the novel appearance of lymph node metastases. Miyauchi *et al.* reported that the appearance of lymph node metastases did not necessarily relate to enlargement of PMCs [13]. Since lymph node metastasis may occur in PMCs that are stable in size, careful examination during active surveillance is mandatory not only for primary lesions but also for regional lymph nodes.

This study has a few limitations. First, this study was designed as a retrospective cohort study. Second, at Kuma Hospital, many sonographers have performed size measurement of PMCs. We did our best to control for sonographer accuracy, but our data may still suffer from interobserver variations that would result in measurement deviations. Since multiple sonographers evaluated patient PMCs, measurement deviations induced by observer variations cannot be completely avoided. Third, follow-up periods were comparably short and the number of sequential ultrasound examinations was relatively low, with a minimum of three examinations pre- and post-PE, indicating that TDR may be to some extent affected by measurement deviations. However, in certain instances, observer variations could occur randomly. If changes in the size of the PMC were due to measurement deviations, the TDR in a proportion of cases would similarly vary in both directions and the TDR in other cases would show no change. However, in our data, as shown in Table 1, TDRs in most cases were >0.1/year at pre-PEs (97.2% for pre-PE-M and 89.3% for pre-PE-V), while TDRs in most cases were <0.1/year at post-PEs (79.5% for post-PE-M and 84.2% for post-PE-V). Additionally, TDRs in a considerably high number of cases were less than −0.1/year at post-PEs (47.4% for post-PE-M and 36.1% for post-PE-V), as also shown in Table 1. These events cannot be explained only by measurement deviations, and we conclude that, despite the presence of measurement deviations, our results regarding change in growth activity between pre- and post-PEs are reliable. Finally, the timing of conversion surgery varied based on the individual case. A proportion of patients preferred conversion surgery even before tumor enlargement occurred. For enlarging PMCs, conversion surgery was performed not only for carcinomas that were 13 mm in size but also for those that showed enlargement by 3 mm. These differences in the timing of conversion surgery may introduce a patient selection bias.

In conclusion, the growth activity significantly decreased in a considerable number of cases, and the tumors shrank in certain cases after enlargement. Therefore, the performance of surgery immediately after attaining PE may be inappropriate. In patients who do not opt for surgery, continuous active surveillance to monitor changes in tumor growth may be considered and may avoid unnecessary surgery. At present, we set the upper limit of tumor size at 13 mm for active surveillance. However, further investigation needs to determine whether this is appropriate or not.

## References

[1] Takami H, Ito Y, Okamoto T, Yoshida A 2011 Therapeutic strategy for differentiated thyroid carcinoma in Japan based on a newly established guideline managed by Japanese Society of Thyroid Surgeons and Japanese Association of Endocrine Surgeons. World J Surg 35:111–121 21042913 10.1007/s00268-010-0832-6

[2] Haugen BR, Sawka AM, Alexander EK, Bible KC, Caturegli P, Doherty GM, Mandel SJ, Morris JC, Nassar A, Pacini F, Schlumberger M, Schuff K, Sherman SI, Somerset H, Sosa JA, Steward DL, Wartofsky L, Williams MD 2016 2015 American Thyroid Association management guidelines for adult patients with thyroid nodules and differentiated thyroid cancer: The American Thyroid Association Guidelines Task Force on thyroid nodules and differentiated thyroid cancer. Thyroid 26:1–133 26462967 10.1089/thy.2015.0020 PMC4739132

[3] Ito Y, Miyauchi A, Kihara M, Kihara M, Higashiyama T, Kobayashi K, Miya A 2014 Patient age is significantly related to the progression of papillary microcarcinoma of the thyroid under observation. Thyroid 24:27–34 24001104 10.1089/thy.2013.0367 PMC3887422

[4] Ito Y, Miyauchi A, Inoue H, Fukushima M, Kihara M, Higashiyama T, Tomoda C, Takamura Y, Kobayashi K, Miya A 2010 An observation trial for papillary thyroid microcarcinoma in Japanese patients. World J Surg 34:28–35 20020290 10.1007/s00268-009-0303-0

[5] Sugitani I, Toda K, Yamada K, Yamamoto N, Ikenaga M, Fujimoto Y 2010 Three distinctly different kinds of papillary thyroid microcarcinoma should be recognized: our treatment strategies and outcomes. World J Surg 34:1222–1231 20066418 10.1007/s00268-009-0359-x

[6] Fukuoka O, Sugitani I, Ebina A, Toda K, Kawabata K, Yamada K 2016 Natural history of asymptomatic papillary thyroid microcarcinoma: time-dependent changes in calcification and vascularity during active surveillance. World J Surg 40:529–537 26581368 10.1007/s00268-015-3349-1

[7] Miyauchi A 2016 Clinical trials of active surveillance of papillary microcarcinoma of the thyroid. World J Surg 40:516–522 26744340 10.1007/s00268-015-3392-y PMC4746213

[8] Miyauchi A, Ito Y, Oda H 2018 Insights into the management of papillary microcarcinoma of the thyroid. Thyroid 28:23–31 28629253 10.1089/thy.2017.0227 PMC5770127

[9] Tuttle RM, Fagin JA, Minkowitz G, Wong RJ, Roman B, Patel S, Untch B, Ganly I, Shaha AR, Shah JP, Pace M, Li D, Bach A, Lin O, Whiting A, Ghossein R, Landa I, Sabra M, Boucai L, Fish S, Morris LGT 2017 Natural history and tumor volume kinetics of papillary thyroid cancers during active surveillance. JAMA Otolaryngol Head Neck Surg 143:1015–1020 28859191 10.1001/jamaoto.2017.1442 PMC5710258

[10] Oh HS, Ha J, Kim HI, Kim WG, Lim DJ, Kim TY, Kim WB, Shong YK, Chung JH, Baek JH 2018 Active surveillance of low-risk papillary thyroid microcarcinoma: a Multi-Center Cohort Study in Korea. Thyroid 28:1587–1594 30226447 10.1089/thy.2018.0263

[11] Sanabria A 2018 Active surveillance in thyroid microcarcinoma in a Latin-American cohort. JAMA Otolaryngol Head Neck Surg 144:947–948 30178005 10.1001/jamaoto.2018.1663 PMC6233831

[12] Miyauchi A, Kudo K, Ito Y, Oda H, Sasai H, Higashiyama T, Fukushima M, Masuoka H, Kihara M, Miya A 2018 Estimation of the lifetime probability of disease progression of papillary microcarcinoima of the thyroid during active surveillance. Surgery 163:48–52 29103582 10.1016/j.surg.2017.03.028

[13] Miyauchi A, Kudo T, Ito Y, Oda H, Yamamoto M, Sasai H, Higashiyama T, Masuoka H, Fukushima M, Kihara M, Miya A 2019 Natural history of papillary thyroid microcarcinoma: kinetic analysis on tumor volume during active surveillance and before presentation. Surgery 165:25–30 30413323 10.1016/j.surg.2018.07.045

[14] Vincent TL, Gatenby RA 2008 An evolutionary model for initiation, promotion, and progression in carcinogenesis. Int J Oncol 32:729–737 18360700

[15] Juan J, Muraguchi T, Lezza G, Sears RC, McMahon M 2014 Diminished WNT -> β-catenin -> c-MYC signaling is a barrier for malignant progression of BRAFV600E-induced lung tumors. Genes Dev 28:561–575 24589553 10.1101/gad.233627.113 PMC3967046

[16] Choi YW, Nam GE, Kim YH, Yoon JE, Park JH, Kim JH, Kang SY, Park TJ 2019 Abrogation of B-RafV^600E^ induced senescence by Fox M1 expression. Biochem Biophys Res Commun 516:866–871 31270027 10.1016/j.bbrc.2019.06.144

